# EEG-Based Identity Authentication Framework Using Face Rapid Serial Visual Presentation with Optimized Channels

**DOI:** 10.3390/s19010006

**Published:** 2018-12-20

**Authors:** Ying Zeng, Qunjian Wu, Kai Yang, Li Tong, Bin Yan, Jun Shu, Dezhong Yao

**Affiliations:** 1 Key Laboratory for NeuroInformation of Ministry of Education, School of Life Science and Technology, University of Electronic Science and Technology of China, Chengdu 610000, China; yingzeng@uestc.edu.cn; 2 China National Digital Switching System Engineering and Technological Research Center, Zhengzhou 450001, China; qunjian1992@163.com (Q.W.); ykfer09@163.com (K.Y.); tonglipku@pku.edu.cn (L.T.); ybspace@hotmail.com (B.Y.); shujun1127@163.com (J.S.)

**Keywords:** identity authentication, EEG, face image, genetic algorithm, rapid serial visual presentation

## Abstract

Electroencephalogram (EEG) signals, which originate from neurons in the brain, have drawn considerable interests in identity authentication. In this paper, a face image-based rapid serial visual presentation (RSVP) paradigm for identity authentication is proposed. This paradigm combines two kinds of biometric trait, face and EEG, together to evoke more specific and stable traits for authentication. The event-related potential (ERP) components induced by self-face and non-self-face (including familiar and not familiar) are investigated, and significant differences are found among different situations. On the basis of this, an authentication method based on Hierarchical Discriminant Component Analysis (HDCA) and Genetic Algorithm (GA) is proposed to build subject-specific model with optimized fewer channels. The accuracy and stability over time are evaluated to demonstrate the effectiveness and robustness of our method. The averaged authentication accuracy of 94.26% within 6 s can be achieved by our proposed method. For a 30-day averaged time interval, our method can still reach the averaged accuracy of 88.88%. Experimental results show that our proposed framework for EEG-based identity authentication is effective, robust, and stable over time.

## 1. Introduction

Identity authentication is an essential safety precaution in many fields, including national security, public security, e-commerce, etc. Conventional authentication systems are based on passwords that are entered with a keyboard, touch screen, or mouse. More recent approaches employ biometric mechanisms, especially approaches based on neural activities (which are currently being explored). Compared to conventional biometrics, such as faces, fingerprints or DNA, brain biometrics [1] offers interesting advantages. Brain biometrics cannot be acquired from a deceased person; furthermore, they are difficult to coerce, since threats of violence or blackmail are very likely to put significant stress on the user, and stress will strongly impact an individual’s brain activities [2]. Electroencephalogram (EEG) signals, which originate from neurons in the brain, have drawn considerable interests from researchers, due to its ease of use, portability, low cost and high temporal resolution [3]. Since an individual’s neural activity pattern is unique [4], it is impossible to imitate one’s mind [5], and circumvent the biometry field [6]; this biometric trait may change the traditional “pass-word” into “pass-thought”.

The first EEG-based identity authentication approach [7] was proposed in the late 90s, then a number of different EEG-based authentication methods were proposed. These methods can be divided into two categories: spontaneous or evoked EEGs, based on the absence or presence of a stimulus. The former includes rest eyes-open/eyes closed (REO/REC), whereas the latter involves visual stimulus presentations, mental tasks, and emotional stimuli. 

In 1999, Poulos et al. [7] developed the first identity authentication system based on EEG signals. The EEG data of 4 users and 75 imposters under REC condition were collected. Auto-regressive parameters and learning vector quantization network were adopted, and the correct recognition rates of 72% and 84% were achieved, respectively. Palaniappan et al. [8] constructed a dataset of VEP signals from 20 subjects. The subjects focused on recognizing stimulus images from the Snodgrass and Vanderwart picture set [9]. The highest accuracy of 92.84% was obtained using the simplified fuzzy adaptive resonance theory. Sun et al. [10] collected the EEG signals of nine subjects while they imagined moving their right or left index finger. The researchers concluded that imagining the movements of the left index finger was more appropriate for identity identification with an accuracy of 95.6%. M. Abo-Zahhad et al. [11] proposed a novel authentication system based on the fused features of EEG and EOG. The lowest verification equal error rates (EERs) were achieved using score fusion for relaxation and VEPs with EERs of 1.3% and 1.41%, respectively, in a database of 22 subjects.

Although these previous works have achieved amazing progress in EEG-based authentication, there are some key issues that needed to be addressed for practical applications. First, current works are mainly based on event-related potentials (ERPs) detection, which usually have to average several trials data to improve the signal to noise ratio, hence the time required for authentication using those methods are relatively long; second, in most works, the neural activities are collected using 32 or more channels of EEG acquisition devices, which makes the set up and preparation inconvenient. Therefore, channel selection and optimization is essential for making the EEG-based identity authentication more feasible for use in daily life. Third, since functional brain activities are constantly changing, one clear question regarding identity authentication based on brain activities is whether it is stable over time. Hence, permanence should be considered as an important evaluation criteria.

The face is a traditional biometric trait for identity authentication [12], and existing studies in cognitive neuroscience have shown that our brain has specific regions when processing face stimulus, and the brain activity response to one’s own face is remarkedly different from the response to familiar or unfamiliar non-self-faces [13,14]. Hence, an EEG-based identity authentication framework using self and non-self-face images is proposed in this study, to combine face and EEG, two kinds of biometric traits. One interesting and meaningful related work was accomplished by Yeom et al. [15]. They used self or non-self-face images as stimuli to evoke subject-specific brain activities based on neurophysiological evidence from both EEG and fMRI. A unique subject-specific brain-wave pattern called visual self-representation was elicited by Yeom’s experimental paradigm. They obtained an average accuracy of 86.1% across 10 subjects using a non-liner support-vector machine. However, the time required for authentication was at least 31.5 s, which makes the system difficult for practical use. In addition, no real imposter was used to test the system’s performance.

To achieve rapid authentication, facial images are presented by rapid serial visual presentation (RSVP) [16] in our paradigm, which can present a large number of stimuli in a short time, eliciting strong event-related potentials (ERPs) [17]. The EEG data that reflect the neural responses to self and non-self-face stimuli are investigated. Significant differences are found under different situations. On the basis of this, an authentication method based on Hierarchical Discriminant Component Analysis (HDCA) [18,19] and Genetic Algorithm (GA) [20,21] was proposed to build subject-specific models with optimized fewer channels. As GA is an adaptive probabilistic search algorithm for global optimization inspired by the laws of natural selection and genetics, the standard parallel GA is used to search for optimized channels. Moreover, the accuracy and stability of the model over time are evaluated to demonstrate the effectiveness and robustness of our method.

## 2. Materials and Methods 

### 2.1. Particpants

Forty-five subjects with a mean age of 22.4 years (standard deviation [SD] = 2.1 years) with normal or corrected to normal visual acuity participated in the experiment. None of the subjects had a history of neurological disease. All subjects gave their informed consent for inclusion before they participated in the study. The study was conducted in accordance with the Declaration of Helsinki, and the protocol was approved by the Ethics Committee of China National Digital Switching System Engineering and Technological Research Center (NDSC-EEG-20180322-03). All subjects received monetary compensation for participating in the experiment.

The subjects were divided into two groups: Users (15 subjects), imposters (30 subjects, two for each user).

### 2.2. Stimuli

Stimuli consists of 195 face images (15 user face images, one for each user; 180 non-user face images, which are collected from student volunteers with their written informed consents for academic research use). All the face images present only facial information with no expression. Each grayscale face image is resized to 400 × 400 pixels. The luminance of the entire stimulus array is measured and do not differ between user face images and non-user-face images.

### 2.3. Experimental Procedure and Design

Stimuli are centrally presented against a black background on a 23-inch computer screen with a refresh rate of 60 Hz at a viewing distance of 80 cm. The experiment is conducted in a quiet environment without light and electromagnetic interference. 

**Figure 1:**
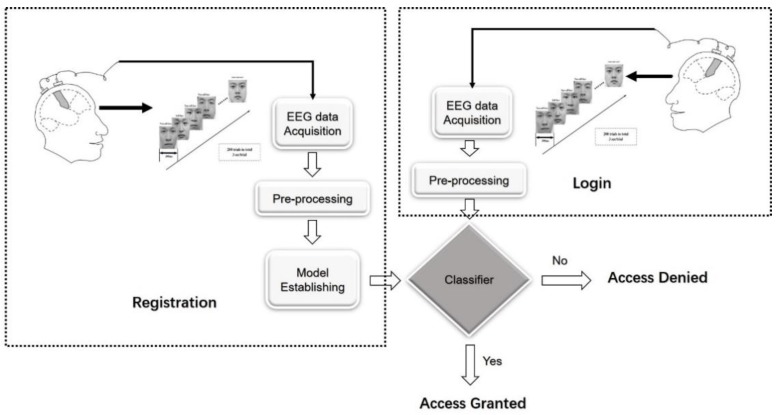
The face image-based RSVP framework for identity authentication.

The face image-based RSVP framework for identity authentication consists of two sections: Registration and login, as shown in Figure 1.

**Figure 2:**
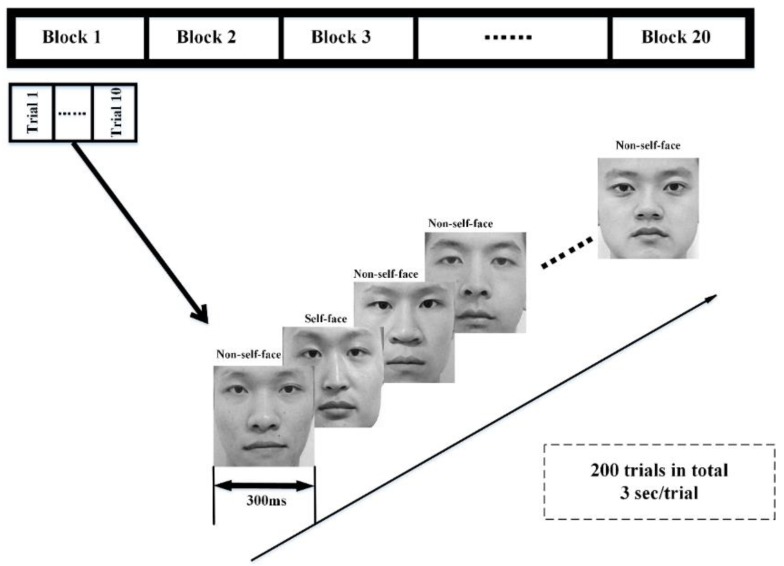
The self and non-self-face RSVP paradigm for identity authentication.

During the registration section, subjects in the user group are regarded as legal users, and a specific face image sequence is designed for each subject in the user group. For each trial, there is one face image of the user as the self-face image and nine face images selected randomly from the non-user face image set as the non-self-face images. The duration time for each image is 300 ms. The presentation order of the self and non-self-face images in each trial is randomized to avoid the effect of subject prediction on the next stimulus. This section consists of 20 blocks, and each block consists of 10 trials (for trials in the same block, the face images are the same but in random orders; for trials in different blocks, the self-face image is the same, while the non-self-face images are different among blocks), as shown in Figure 2. A short rest comes after 10 blocks. Subjects are asked to focus on the specific face image sequence, while their EEG signals are collected synchronously and an authentication model for each specific user is generated using these signals. 

During the login section, to login into a legal user’s account, subjects should watch the specific face image sequence corresponding to the user, while their EEG signals are collected. 

### 2.4. Electrophysiological Recording

The subjects were seated individually in an electrically shielded, dimly lit room. EEG data were measured when the subjects were watching face image sequences during registration or login section. EEG data were recorded using a g.USBamp amplifier with 16 wet active electrodes positioned according to the International 10–20 system and digitized with the sample rate of 2400 Hz. The 16 channels used were as follows: Fz, Cz, P3, Pz, P4, Po7, Oz, Po8, C3, C4, F3, F4, Af7, Af8, Cp5, and Cp6. During the entire experimental task, the impedances of electrodes was kept below 5 kΩ.

The entire EEG dataset acquisition includes two sessions. In session 1, EEG datasets are obtained to generate and test the capability of the identity authentication model. In session 2, EEG datasets are acquired to test the permanence capability of the model.

During session 1, the 15 subjects in the user group are considered as the legal users; hence, specific face image-based RSVP sequences are designed for each user and shown to them. Users were told to focus on their own face images and count the number of occurrences in their minds. To generate and test the performance of the authentication model for each user, two different fraud scenarios are considered. In the first scenario (blind invasion), a subject is regarded as an imposter when he does not know the invasion account; hence, the subject will watch the specific face image sequence without knowing which face image is that of the legal user. In the second scenario (non-blind invasion), a subject is told to invade a certain user’s account. Thus, when the specific face image sequence is presented, the subject will know which face image is of the legal user and will try to invade using the same strategy of the user. For each legal user, we collected two imposters’ EEG data, one for blind invasion, the other for non-blind invasion.

During session 2, to evaluate the permanence of the model, EEG datasets are acquired for each subject in the user group, with an average time interval of 30 days from the first acquisition. The permanence test is performed on the classifiers generated from session 1.

### 2.5. Data Processing

#### 2.5.1. EEG Data Preprocessing

The raw EEG data were re-referenced using REST [22,23], and were then filtered by a low-pass Chebyshev digital filter with a passband of 40 Hz and a stopband of 49 Hz for further analysis. The data were downsampled from 2400 Hz to 600 Hz by averaging four consecutive samples. Epochs of 200 ms before to 1000 ms after each user’s face image onset were extracted and were baseline corrected by subtracting the averaged amplitude of a small data segment (200 ms to 0 ms) before stimulus onset from each data point.

#### 2.5.2. Classification with Hierarchical Discriminant Component Analysis (HDCA)

There are significant differences between the EEG signals induced by self and non-self-face stimulus, as shown in our previous studies [24,25]. Hence, it is feasible to authenticate a person’s identity by determining whether the EEG signals are induced by self-face image or not. However, the EEG data always contain a certain degree of external noise, and their spatial distribution, amplitudes and latencies may vary with subjects.

Hierarchical discriminant components analysis (HDCA) [18,19] is a classic method using both spatial and temporal information of the EEG signals for classification. In this work, HDCA is used to classify the specific EEG signals evoked by self-face and non-self-face image, which extracts both spatial and temporal features of the signals. The details of the algorithm are as follows:

(1) Spatial features extraction: 

It is assumed that the spatial distribution of the EEG activities changes over time with a temporal resolution (T) of 100 ms. Hence, weight vectors, $w_{n,i}$, are needed to be found for several 100 ms windows following each face image presentation. Here, Fisher linear discriminant (FLD) analysis is used to calculate the spatial coefficients, $w_{n,i}$. First, each channel of the EEG signals is divided into *N* segments on average by the given time window (100 ms). Then, the weight for each channel is calculated in each time window to maximize the difference between EEG signals evoked by self-face and non-self-face images, using the Fisher linear discriminant analysis. Therefore, the multichannel EEG signals are compressed into a single channel signal, namely:

(1) $$y_{n}=\sum_{i}w_{n,i}x_{i,n}\;i=1,2,3\ldots 16$$

where *i* and *n* denotes the number of channels and EEG segments, respectively; $x_{i,n}$ and $w_{n,i}$ represent the *i*-th channel EEG signal in *n*-th segment and its weights; and $y_{n}$ is the desirable single channel signal.

(2) Temporal feature extraction: 

The obtained single channel signal $y_{n}$ for each of the separated time windows are first average by time point. Then, the resulting values for each of the separate time windows $y_{k}$ are then combined in a weighted average to provide a final interest score $Y_{S}$ for each image, namely:

(2) $$Y_{S}=\sum_{k}v_{k}y_{k}$$

The weight $v_{k}$ of $y_{k}$ are calculated to make the self-face score higher than the non-self-face score by using the logistic regression method.

#### 2.5.3. Channel Selection with Genetic Algorithm (GA)

Feature selection and channel optimization is an essential step for identity authentication based on EEG. Actually, models built based on optimized channels for each user may not only help improve the accuracy but also the robustness for identity authentication.

Genetic Algorithm (GA) is an adaptive probabilistic search algorithm for global optimization, inspired by the laws of natural selection and genetics. GA follows the natural evolutionary model, and starts with an initial population of individuals, which consists of a fixed length continuous or discrete strings analogous to the chromosomes in a DNA. Each individual represents a possible solution to a given optimization problem and over successive generations evolves toward a set of more optimal or fit individuals. In this study, GA is used to optimize the channels for identity authentication. Channel is regarded as a unit, which means that features from a certain channel are taken as a whole to be reserved or removed together. The details of the algorithms are as follows:

(1) Initial population generation

Initial population of individuals is generated randomly as a set of binary sequences. Each sequence is of length 16, in which each binary bit denotes if the corresponding channel is chosen or not. Bit ‘1’ means chosen, while bit ‘0’ means not chosen. The size of the initial individuals is set to be 100.

(2) Fitness evaluation

Each individual in the population is assigned a fitness value based on a fitness function, which evaluates how good a solution is to the problem. In our work, fitness is evaluated on the basis of the classification accuracy by HDCA. 

(3) Judge with optimization criteria 

Optimization criteria identifies if the pre-set generation is reached, or the fitness value is no longer rising. If the criteria are satisfied, then the algorithm ends; if not, a new generation of individual is produced and the algorithm goes to step 2. The evolution process consists of three basic operations: selection, crossover, and mutation. The probability of crossover operation is set to 0.85, while the probability of mutation operation is set to 0.1.

## 3. Results

### 3.1. Face Image Evoked ERPs Anaylses

ERP analyses were used to illustrate the variability of the neural responses induced by the face image sequences. EEG data were processed and analyzed using EEGLAB. Artifact free data were epoched—200 to 1000 ms around the users’ face images. Hence, for each subject, there are 200 available trials. For each subject in the user group, the EEG data are induced by self-face images, while for their two corresponding imposters, the EEG data are induced by non-self-face images. 

**Figure 3:**
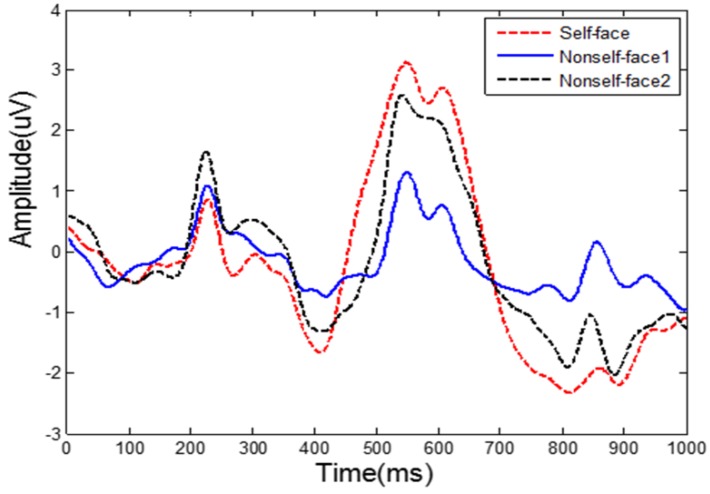
The grand ERPs evoked by self-face and non-self-face at electrode P4.

In order to assess brain activities evoked by self-face and non-self-face images, averaging across all trials in each condition were done. Figure 3 shows the grand ERPs evoked by self-face and non-self-face in two scenarios at electrode P3. The red dotted line shows the averaged ERP across all trials and users, when their own face images were presented. The black dotted and the blue solid lines are the averaged ERP evoked by non-self-face in two different scenarios. The blue solid line shows the averaged ERPs across all trials and imposters who do not know the users. The black dotted line shows the averaged ERPs across all trials and imposters who are familiar to the users. This figure shows that the ERPs (N170, N250, P3a, and P3b) evoked by the same face image are different under the three conditions. The amplitude of N250 for the users are little larger than for the imposters. The difference of P3a and P3b among the three conditions is the largest. The amplitude of P3a and P3b for the users are much larger than those for the imposters, especially for the imposters in Scenario 1.

**Figure 4:**
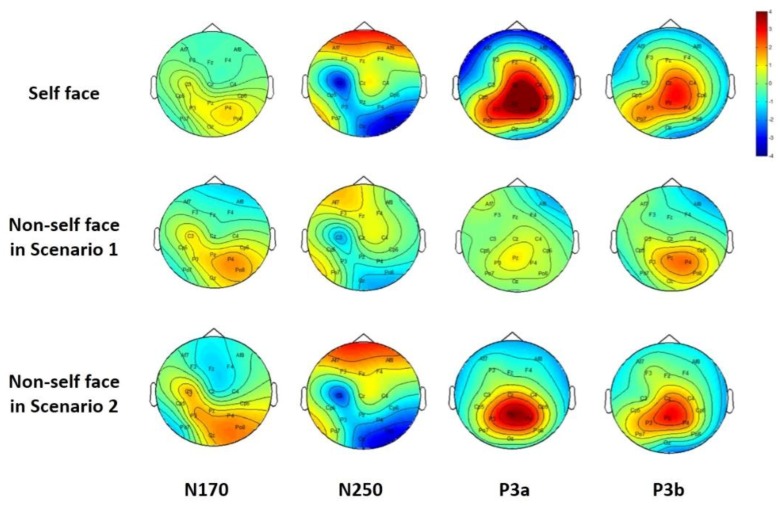
The topographic maps of EEG data for self-face and non-self-face.

In addition, the topographic maps under the three conditions are compared, as shown in Figure 4. Significant difference can be found for self-face and non-self-face in Scenario 1 at P3a and P3b. Although the two maps of self-face and non-self-face in Scenario 2 are similar at P3a and P3b, their strengths are different. More strong signals are evoked for users when their own face images are presented. 

### 3.2. Identity Authenication Performance

There were 45 subjects in total, divided into two groups (user group and imposter group). In the user group, there were 15 subjects. For each user, there were two corresponding imposters (one had the knowledge of who he intends to invade, the other did not). For these three subjects, artifact free and baseline corrected data were epoched 0 to 1000 ms around the user’s face image onset. There were 200 trials data for each subject, which is 16 × 600 × 200 (16 channels, 600 time points, 200 trials). To obtain a better signal-to-noise ratio, the data was averaged across each two adjacent trials. Hence, for each subject, the sample data is 16 × 600 × 100 (16 channels, 600 time points, 100 trials). There were 45 samples, which included 15 samples for self-face images and 30 samples for non-self-face images.

Classification accuracy (ACC), false acceptance rate (FAR), and false rejection rate (FRR) was used to evaluate the performance of the system for each user, which are defined as follows:

(3) $$\mathrm{ACC}=\frac{\mathrm{number}\;\mathrm{of}\;\mathrm{correctly}\;\mathrm{authenticated}\;\mathrm{samples}}{\mathrm{total}\;\mathrm{number}\;\mathrm{of}\;\mathrm{test}\;\mathrm{samples}}$$

(4) $$\mathrm{FAR}=\frac{\mathrm{number}\;\mathrm{of}\;\mathrm{falsely}\;\mathrm{accepted}\;\mathrm{samples}}{\mathrm{total}\;\mathrm{number}\;\mathrm{of}\;\mathrm{imposter}\;\mathrm{test}\;\mathrm{samples}}$$

(5) $$\mathrm{FRR}=\frac{\mathrm{number}\;\mathrm{of}\;\mathrm{falsely}\;\mathrm{rejected}\;\mathrm{samples}}{\mathrm{total}\;\mathrm{number}\;\mathrm{of}\;\mathrm{user}\;\mathrm{test}\;\mathrm{samples}}$$

Authentication performance is explored by performing two classification tests. In the first test, a 5-fold cross validation is used to determine the authentication performance for all classification methods applied in this study. The 45 samples collected in session 1 are used in this test. The second test is the permanence test: for each user, the EEG data collected in session 2 are tested on the classifiers generated from session 1. Thus, only classification accuracy (ACC) is adopted to evaluate the performance.

The classification results by HDCA and GA optimized HDCA (GA-HDCA) are shown in Table 1. It can be seen from the results that the GA-optimized HDCA algorithm had a better performance in ACC, FAR and FRR than the traditional HDCA algorithm. The average classification accuracy (ACC) is improved from 89.16% to 94.26%, whereas the false acceptance rate (FAR) and the false rejection rate (FRR) decrease from 10.97% to 6.27%, and 10.77% to 5.26%, respectively.

In order to test the permanence of the proposed GA-HDCA algorithm, a second EEG data acquisition session was conducted for each user with a 30-day average time interval. For each user, the EEG data collected in the second session was tested on the classifiers generated from the first session. Table 2 shows the permanence test results for each user using HDCA and GA-HDCA. For a 30-day average time interval, the model generated by the proposed GA-HDCA algorithm can still achieve an averaged accuracy of 88.88%, better than HDCA. The results demonstrate the stability of our method, which is essential for practical applications of the EEG-based identity authentication system.

### 3.3. Optimized Channels for Authentication

Channel optimization for identity authentication mainly serves two purposes. One is to enhance practicality. Using fewer channels in EEG-based identity authentication system will greatly reduce preparation time and make the system more convenient. The other is to improve authentication performance. Since there are significant differences among individuals’ EEG data, even under the same condition, selecting specific channels for each user will not only improve classification accuracy, but also make the model specific for the user and more robust to resist invasion. Table 3 shows the optimized channels selected by the proposed GA-HDCA for the 15 users. “0” denotes that the corresponding channel is not chosen, while “1” denotes that the corresponding channel has been chosen. It can be seen that the selected channels are different among users. The selected times of each channel are also calculated. The most selected electrodes are “P4”, “Pz”, and “P3”, where the “P4” electrode is selected 14 times out of 15 users.

## 4. Discussion

Evoking significant and stable individual specific features is essential for identity authentication. The face is a traditional biometric trait that can well represent a person’s identity, and EEG signals can reflect a person’s brain activities. Hence, a face image-based RSVP paradigm for identity authentication is proposed, which combines the face and EEG, the two kinds of biometric traits. Experimental results have shown that EEG signals are different when a person watches his own face and another person’s face. Although the self-face image and non-self-face image can both evoke N170, N250, and P300 components, their amplitude, latency and spatial distribution are different between the two categories, especially for the P300 component. Even for an imposter who is familiar with the user he intends to invade, the amplitude and the latency of the P300 component evoked by the non-self-face is lower and longer than that of the self-face. The lower amplitude may indicate that non-self-face is less sensitive for a person than self-face, while the longer latency may reflect more complicated processing when dealing with non-self-face than self-face. 

Compared to the meaningful related work accomplished by Yeom et al. [15], in our study, RSVP paradigm is induced into an authentication framework, which enables us to present a stimulus in a very short time (300 ms). Averaged authentication accuracy of 94.26% within 6 s can be achieved in our study. Both real-time capability and accuracy of the system are significantly improved, compared to Yeom’s work.

**Figure 5:**
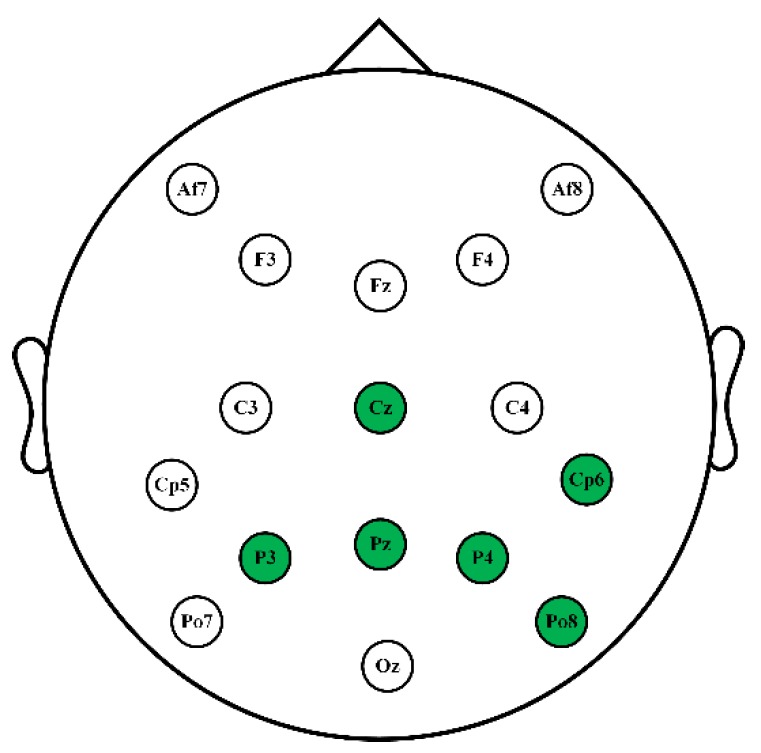
The most related electrodes for face image-based RSVP paradigm.

On the basis of HDCA, a GA-optimized HDCA (GA-HDCA) method is proposed to build subject-specific models for classifying user and imposter, using the differences between self-face and non-self-face evoked EEG signals. The GA-HDCA tries to find the global optimization solution for selecting the best set of channels in classifying the two category of EEG signals. Results show that the solution is subject-specific. After channel optimization, the overall authentication performance is improved in ACC, FAR and FRR, which means that the optimization can not only reduce redundant information and increase accuracy, but it also makes the model subject-specific to resist invasion. Figure 5 shows the positions of the most related electrodes for our proposed authentication framework based on face image-RSVP. These six electrodes are the most selected ones by GA-HDCA across the 15 users. This result is consistent with our previous study [24,25], where “Cz”, ”Pz”, “P3”, and “P4” were also found to be important for classification. Moreover, these selected electrodes are exactly distributed in the brain regions where the EEG signals evoked by self-face and non-self-face images are most different, especially for the P300 component. 

A comparison of our method with previous related works is provided in Table 4. The superiority of our proposed method can be seen from the performance comparison. For our proposed method, the average accuracy of 94.36% is the highest, whereas the FAR of 6.27% and FRR of 5.26 are the second lowest. Moreover, the stability of our method is also tested using data collected with an averaged interval of 30 days. It can still achieve a promising accuracy of 88.88%. However, since the proposed method is attempting to find the global optimized solution, for each individual, the time required for training the model with GA-HDCA is approximately 40 min, 10 times longer than that of HDCA. But once the model has been built, it is ready for use. Thus, this time requirement will only affect the registration, not the login section. Hence, this does not affect the proposed method for practical use. Furthermore, in order to make our system applicable to real-time scenarios, the authentication model for each user is trained using data of two adjacent trials. This means that during the login section, the model only needs the data of two trials to determine whether the person is a legal user or an imposter. Since the duration time for each trial is 3 s, the time required for one authentication is only 6 s in our system, which shows better real-time performance than previous studies. 

## 5. Conclusions

In this paper, a face image-based RSVP paradigm is designed for identity authentication, which combines face and EEG, the two kinds of biometric traits, to evoke more specific and stable traits for authentication. Significant differences are found for the ERP components and topographic maps induced by self-face and non-self-face (familiar and not familiar). On the basis of this, an authentication method based on HDCA and GA is proposed to build subject-specific models with optimized fewer channels. The averaged authentication accuracy of 94.26% in 6 s with averaged 6 channels is achieved by our proposed method. For a 30-day averaged time interval, our method can still reach the averaged accuracy of 88.88%. Hence, the experimental results show the effectiveness, robustness, and stability of the proposed framework for EEG-based identity authentication. In future work, the experiment will be repeated with a longer interval time to further explore the system’s stability. Portable EEG acquisition equipment, such as the Emotiv EPOC headset, will be used to improve system practicability. Moreover, the open set ability of our system will be tested with random people who are not used in the training phase.

## Figures and Tables

**Table 1:** Authentication performances with HDCA and GA-HDCA.

User	ACC (%)		FAR (%)		FRR (%)	
	HDCA	GA-HDCA	HDCA	GA-HDCA	HDCA	GA-HDCA
1	82.5	92.8	21.5	9.5	13.5	5.0
2	92.3	95.0	05.5	2.5	10.0	7.5
3	86.3	92.0	13.0	11.0	14.5	5.0
4	93.8	97.5	05.5	3.5	7.0	1.5
5	86.8	93.3	13.0	6.0	13.5	7.5
6	89.0	96.3	12.0	3.5	10.0	4.0
7	85.8	93.0	11.5	5.5	17.0	8.5
8	91.0	97.3	12.5	5.0	5.5	0.5
9	91.5	94.8	6.0	4.0	11.0	6.5
10	85.0	93.3	16.5	8.0	13.5	5.5
11	93.0	97.0	6.0	1.5	8.0	4.5
12	92.8	92.8	6.0	9.0	8.5	5.5
13	91.5	95.8	9.5	4.0	7.5	4.5
14	85.8	92.0	19.5	14.0	9.0	2.0
15	90.3	91.0	6.5	7.0	13.0	11.0
Mean (std)	89.16 (3.52)	94.26 (2.12)	10.97 (5.22)	6.27 (3.47)	10.77 (3.27)	5.26 (2.74)

**Table 2:** The classification accuracy of each user with a 30-day averaged time interval.

User	ACC (%)	ACC (%)
	HDCA	GA-HDCA
1	80.2	86.9
2	72.9	80.7
3	88.5	85.1
4	89.5	95.6
5	86.9	85.8
6	84.9	92.2
7	86.8	83.1
8	91.9	94.0
9	78.8	80.4
10	84.6	89.9
11	94.0	93.0
12	88.1	91.4
13	82.2	86.2
14	94.1	96.6
15	89.7	92.3
Mean (std)	86.21(5.83)	88.88(5.25)

**Table 3:** The optimized channels for all the 15 users.

User	Channels																Number of Selected Channels
	Fz	Cz	P3	Pz	P4	Po7	Oz	Po8	C3	C4	F3	F4	Af7	Af8	Cp5	Cp6	
**1**	0	0	1	1	1	0	0	1	0	0	0	0	0	0	0	0	4
**2**	0	0	0	1	1	0	1	0	0	1	0	0	0	0	0	1	5
**3**	0	0	1	1	1	0	0	1	0	0	0	0	0	0	0	0	4
**4**	0	0	1	1	1	0	0	1	0	0	0	0	0	0	1	0	5
**5**	0	1	1	0	1	0	0	1	0	0	0	0	0	0	1	0	5
**6**	0	0	1	1	1	0	0	0	0	0	0	0	0	0	0	0	3
**7**	0	1	0	1	0	0	0	0	1	0	0	0	0	0	0	1	4
**8**	0	0	1	1	1	0	0	0	1	1	0	0	0	0	1	1	7
**9**	0	1	0	0	1	0	0	1	0	1	0	0	0	0	0	1	5
**10**	0	1	1	1	1	0	1	0	1	0	0	0	0	0	1	0	7
**11**	0	1	0	0	1	0	0	1	0	1	0	1	0	1	0	0	6
**12**	1	1	1	0	1	1	1	1	0	0	0	0	1	0	0	1	9
**13**	0	0	0	1	1	0	1	1	1	1	0	0	0	0	1	1	8
**14**	0	1	1	1	1	0	1	0	1	0	0	0	0	0	0	1	7
**15**	0	0	1	1	1	0	1	1	1	0	1	1	0	1	1	1	11

**Table 4:** Performance comparison with previous related works.

Author	Stimulus Type	Time Required (s)	Imposter Scenarios	Stability Test	ACC (%)	FAR (%)	FRR (%)
Armstrong et al. [26]	Text reading	NA	None	Yes	89	NA	NA
Yeom et al. [15]	Self-or non-self-face images	31.5~41	None	None	86.1	13.9	13.9
Marcel et al. [27]]	Motor imagery	15	None	None	80.7	14.4	24.3
Miyamoto et al. [28]	Resting state	60	None	None	79.0	21.0	21.0
Mu et al. [29]	Self- and non-self-photos	6.5	None	None	87.3	5.5	5.6
Wu et al. [25]	Face RSVP	6	2 scenarios	Yes	91.46	9.23	7.85
Proposed method	Face RSVP	6	2 scenarios	Yes	94.26	6.27	5.26
